# Comparative Evaluation of the Properties of Irreversible Hydrocolloid Materials Following the Addition of Two Different Local Anesthetic Agents: An In Vitro Study

**DOI:** 10.7759/cureus.112112

**Published:** 2026-07-05

**Authors:** Vishnuvardhan Venkatachalam, Mathew Chalakuzhiyil Abraham, Vidhyasankari N, Abhinayaa Suthagar, Sebeena Mathew, Shrinnivasan Parameshwaran

**Affiliations:** 1 Prosthodontics and Crown and Bridge, KSR Institute of Dental Science and Research, Tiruchengode, IND; 2 Prosthodontics, KSR Institute of Dental Science and Research, Tiruchengode, IND; 3 Conservative Dentistry and Endodontics, KSR Institute of Dental Science and Research, Tiruchengode, IND; 4 Prosthodontics, Nandha Dental College and Hospital, Tiruchengode, IND

**Keywords:** compressive strength, gag reflex, irreversible hydrocolloids, local anesthetics, tear strength

## Abstract

Aim: This study aims to evaluate the impact of incorporating irreversible hydrocolloid impression materials with local anesthetic agents, specifically 2% lignocaine and 0.5% bupivacaine, on their compressive strength, tear strength, and molecular properties, which can determine their efficacy in managing the gag reflex during impression-making without compromising material performance. Materials and methods: This study evaluated the effects of lignocaine and bupivacaine on the compressive and tear strength of alginate materials. A total of 120 samples were divided into control and experimental groups using Tropicalgin and Algitex, with experimental groups incorporating 2% lignocaine or 0.5% bupivacaine. Strength tests were conducted using standard molds and a universal testing machine. Compressive strength was tested at 50 mm/min under 100 N, and tear strength as load per thickness at 500 mm/min. Results highlight the impact of anesthetic additives on alginate properties. Results: This study showed no statistically significant difference in compressive strength between the control and study groups and a negligible difference in tear strength. A Fourier transform infrared spectroscopy analysis showed optimal molecular affinity between irreversible hydrocolloids and local anesthetic agents (2% lignocaine and 0.5% bupivacaine) across all samples, both before and after setting. Conclusions: This in vitro study, within its limitations, found that adding local anesthetic agents to irreversible hydrocolloid impression materials may reduce the gag reflex without affecting their compressive strength, tear strength, or chemical properties.

## Introduction

In prosthetic dentistry, irreversible hydrocolloid impression materials are frequently used. These hydrocolloids are elastic materials used to create extraoral and intraoral studies and diagnostic casts for individuals who are either completely or partially edentulous [1].

Alginate offers numerous benefits, including low cost, improved patient comfort, ease of use, quick execution, simple technique, hydrophilic nature, and the ability to capture detailed impressions (even with undercuts) in a single step. Disadvantages include poor dimensional stability and limited shelf life. Distortion could occur covertly if the impression material is not kept steady during setting. Since tear strength is lower, thin sections might tear upon removal [2].

The gag reflex acts as a defense mechanism that protects the throat and airway from the entry of foreign objects. Gagging occurs in response to internal or external stimuli that cause the pharynx to contract quickly to protect the airway. Many people experience discomfort during dental treatment due to an excessive gag reflex, making treatment challenging or impossible to complete the procedure [3].

Gag reflexes can be managed or prevented by pharmacological and non-pharmacological techniques. Pharmacological treatments involve the use of peripheral- or central-acting drugs. Topical and local anesthetic agents are examples of peripherally acting drugs. Antihistamines, sedatives, tranquilizers, parasympatholytics, and CNS depressants are some of the groups for centrally acting substances. Non-pharmacological interventions include behavioral modifications and other approaches, such as transcutaneous electrical nerve stimulation, placing salt on the tip of the tongue, use of prosthetic appliances and earplugs, and acupressure and acupuncture techniques [4-6].

Among these, the use of local and topical anesthetics is considered an effective method for reducing excessive gagging [4]. Local anesthetic agents most commonly used in dentistry include articaine, bupivacaine hydrochloride, lignocaine, mepivacaine, and prilocaine, which belong to the amide class [7,8].

Administration of local anesthetic agents around the posterior palatine foramen and the glossopharyngeal nerve block technique was also effective in patients with exaggerated gag reflexes. The deposition of local anesthetic agents may be impossible because they can provoke gagging and result in inaccurate impressions due to tissue distension [9]. Intravascular administration of the anesthetic is the main complication of a glossopharyngeal nerve block, potentially causing toxicity. Another serious issue is upper airway obstruction from excessive anesthesia, which affects the vagus nerve. Mild dyspnea may also occur and can be treated conservatively [9].

Understanding the effect of lignocaine on the properties of irreversible hydrocolloid impression materials is essential. A study by Qaisar et al. reported an insignificant reduction in the compressive strength and tear strength of irreversible hydrocolloid impression material after the addition of lignocaine [10]. The present study aims to evaluate and compare the properties of irreversible hydrocolloid impression materials when supplemented with local anesthetic agents such as lignocaine (2%) and bupivacaine (0.5%). The primary hypothesis of this study is that the addition of local anesthetic agents (2% lignocaine and 0.5% bupivacaine) to irreversible hydrocolloid impression materials will not significantly reduce their compressive and tear strengths below the clinically acceptable limits set by the American Dental Association (ADA) Specification No. 18.

## Materials and methods

Study design and sample selection

An in vitro experimental study was designed to evaluate the physical properties of modified irreversible hydrocolloids. Convenience sampling was used to prepare the specimens in a controlled laboratory setting. All sample mixing and preparation were performed by a single, calibrated operator to eliminate handling variability. The procedures were conducted under strictly controlled environmental conditions, maintaining an ambient laboratory temperature of 23 ± 1°C and a relative humidity of 50 ± 10%, in accordance with standard dental material testing protocols.

The sample size was estimated using the standard formula for comparing continuous variables across multiple groups:

$n = \frac{2\sigma^2 \left(Z_{1-\alpha/2} + Z_{1-\beta}\right)^2}{d^2}$

where $\sigma$ represents the pooled standard deviation from previous literature, and $Z_{1-\alpha/2}$ represents the standard normal variate for the level of significance (set at 1.96 for $\alpha = 0.05$, $Z_{1-\beta}$ represents the standard normal variate for power (set at 0.84 for 80% power), and d represents the expected clinically significant difference. This theoretical calculation was corroborated using G*Power software (Heinrich Heine University Düsseldorf, Düsseldorf, Germany), which indicated a requirement of 10 samples per group to achieve adequate statistical power, yielding a total of 120 samples across the 12 experimental and control groups.

Materials used

The materials used in this study included Tropicalgin (Zhermack; Veneto, Italy), Algitex (Dental Products of India, New Delhi, India), 2% lignocaine hydrochloride (Indoco Remedies, Mumbai, India), and 0.5% bupivacaine hydrochloride (Neon Laboratories, Mumbai, India).

Sample grouping

The sample groupings and distributions are outlined in Table 1.

**Table 1:** Group distribution and methodology

Material	Control (A) (15 mL water)	Experimental (B) (12.8 mL water + 2.2 mL lignocaine)	Experimental (C) (12.8 mL water + 2.2 mL bupivacaine)
Tropicalgin (compressive)	Group IA (n = 10)	Group IB (n = 10)	Group IC (n = 10)
Algitex (compressive)	Group IIA (n = 10)	Group IIB (n = 10)	Group IIC (n = 10)
Tropicalgin (tear)	Group IIIA (n = 10)	Group IIIB (n = 10)	Group IIIC (n = 10)
Algitex (tear)	Group IVA (n = 10)	Group IVB (n = 10)	Group IVC (n = 10)

**Figure 1:**
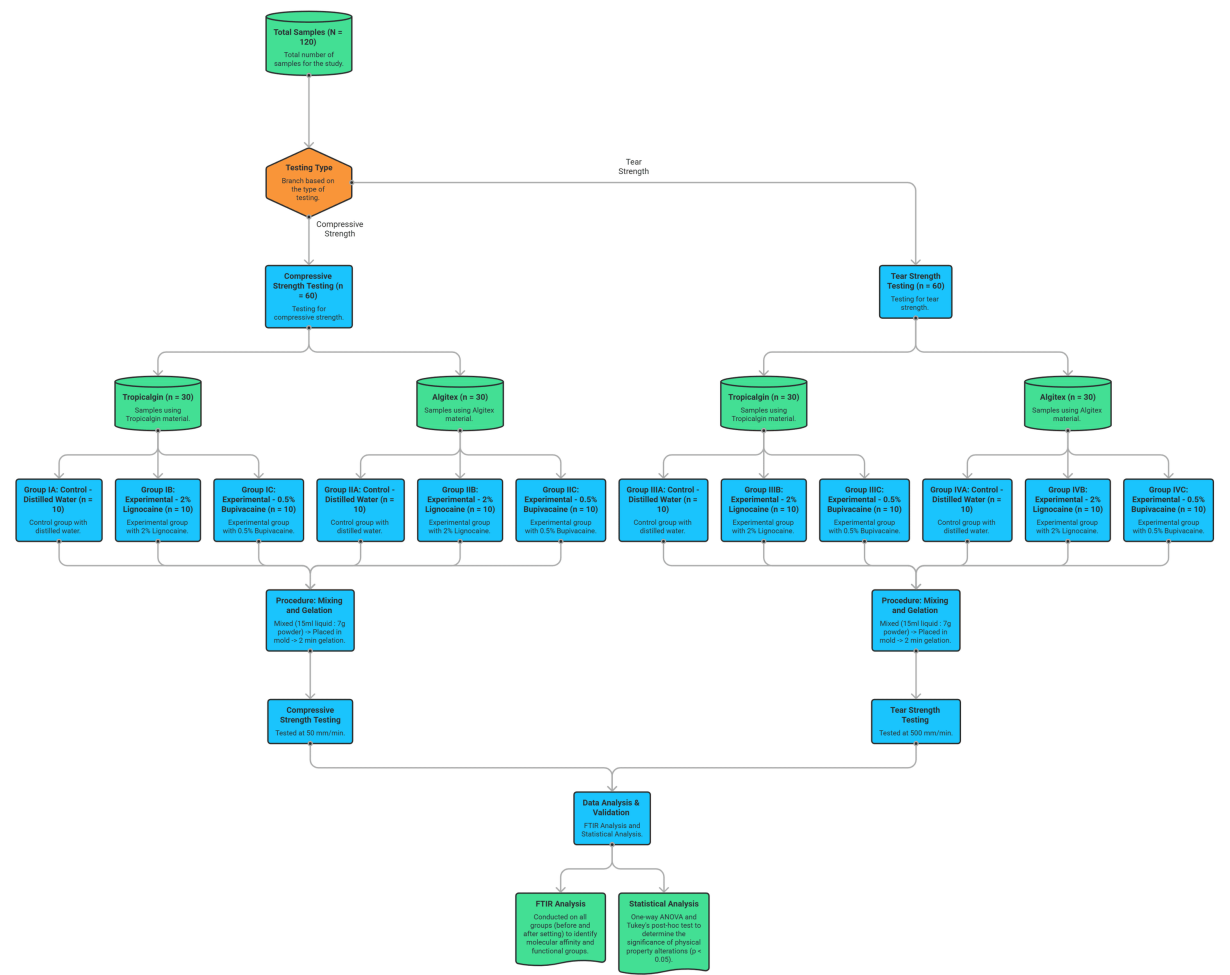
Flow diagram illustrating the study design, sample distribution, testing parameters, and analysis protocol FTIR: Fourier transform infrared spectroscopy

A detailed breakdown of the experimental workflow and sample distribution is illustrated in Figure 1.

To ensure that any variation in compressive or tear strength could be attributed solely to the chemical addition of local anesthetic agents rather than to operator-induced artifacts, a rigorous sample selection process was implemented based on visual inspection and structural integrity.

Only completely gelled, homogeneous specimens prepared according to the manufacturer's recommended powder-to-liquid ratio, free of visible porosity, and successfully retrieved intact from the standardized molds were included in the testing phase. Specimens exhibiting incomplete gelation, visible voids, internal air bubbles, surface irregularities, or marginal tears upon retrieval from the molds were discarded and excluded from the analysis.

Compressive strength

**Figure 2:**
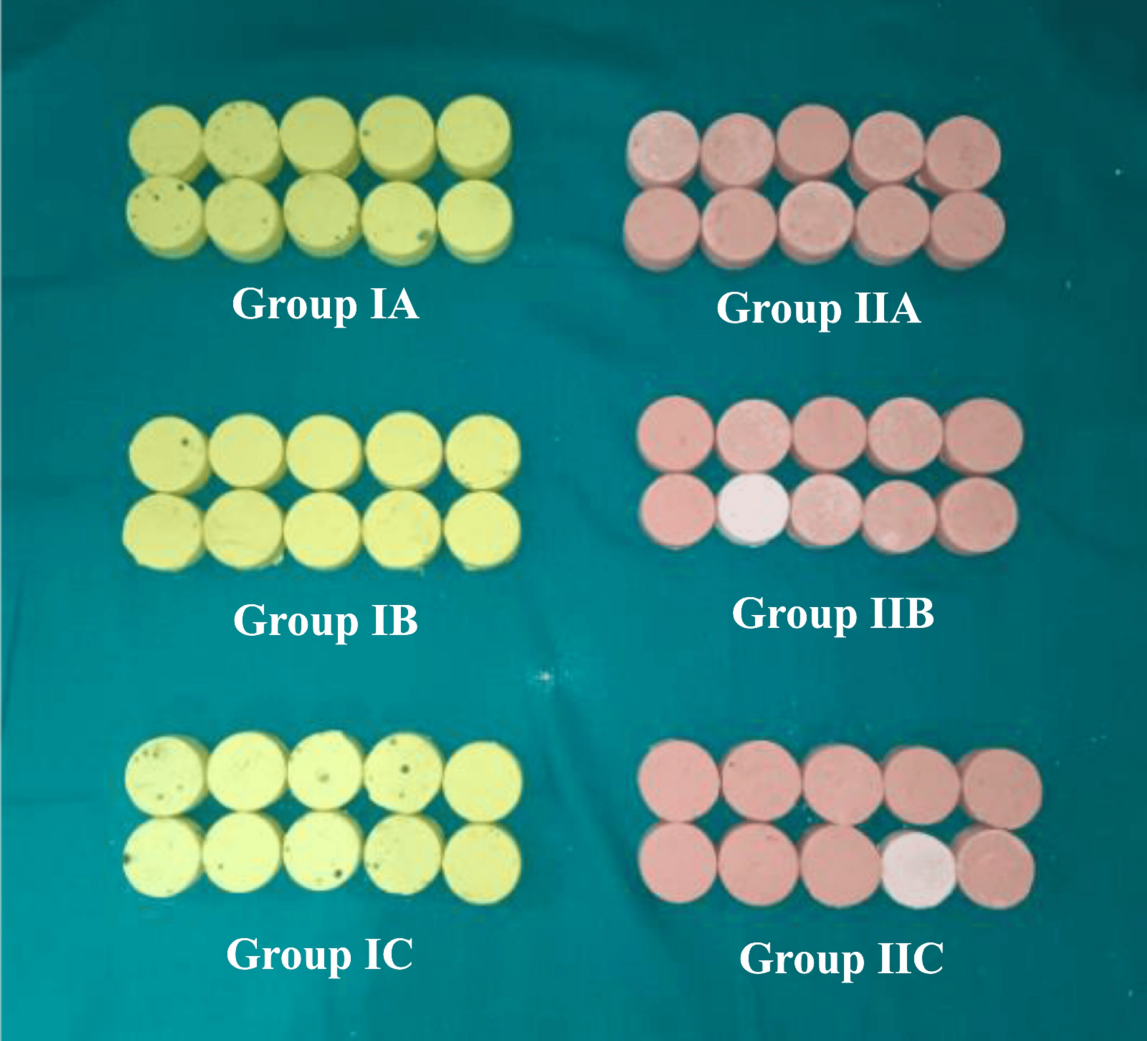
Samples (compressive strength)

**Figure 3:**
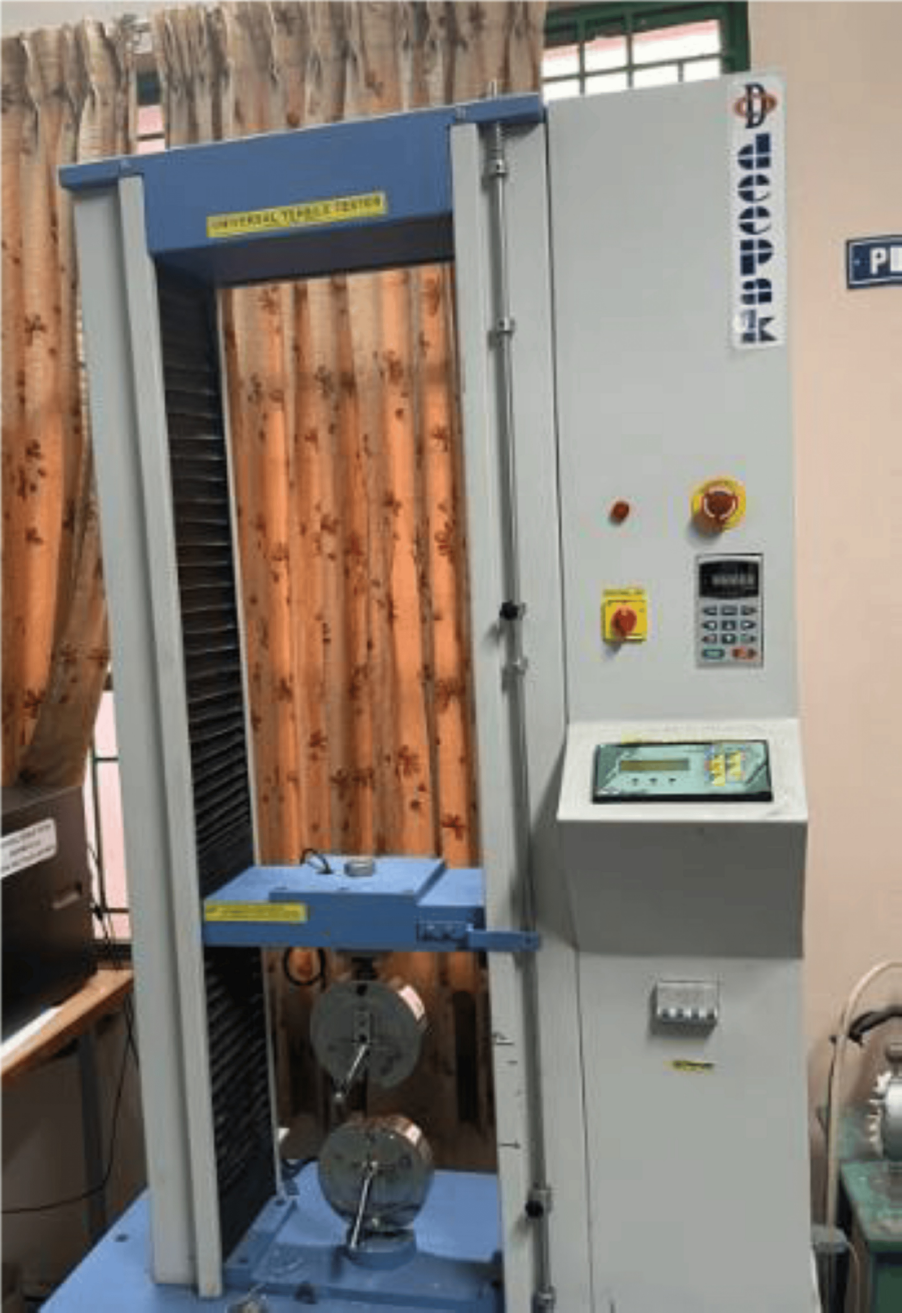
Universal testing machine

For the control group, 15 mL of water was used for mixing with 7 gm of Tropicalgin powder (Group IA), and 15 mL of water was used for mixing with 7 gm of Algitex powder (Group IIA) and then inserted into a cylindrical-shaped mold (Figure 2) (30 x 16 mm); pressure was then manually applied until the two glass slabs were in contact with the mold to ensure an even thickness of alginate during gelation. After two minutes, the cylindrical-shaped alginate specimens were retrieved from the mold and subjected to a compressive strength test immediately, using a universal testing machine (Deepak Poly Plast Pvt. Ltd., Ahmedabad, Gujarat, India) (Figure 3) at a 50 mm/min crosshead speed and 100 N force [11].

For the experimental group, 2.2 mL of lignocaine (2%) and 12.8 mL of water were used for mixing with 7 gm of Tropicalgin powder (Group IB), and 2.2 mL of lignocaine (2%) and 12.8 mL of water were used for mixing with 7 gm of Algitex (Group IIB) and then inserted into a cylindrical-shaped mold (Figure 2) (30 x 16 mm); the pressure was then manually applied until the two glass slabs were in contact with the mold to ensure an even thickness of alginate during gelation. For the other experimental group, instead of lignocaine (2%), bupivacaine (0.5%) was used (Group IC, Group IIC), and the same procedures were followed. After two minutes, the cylindrical alginate specimens were retrieved from the mold and immediately subjected to a compressive strength test using a universal testing machine (Figure 3) at a crosshead speed of 50 mm/min and a force of 100 N [11].

Tear strength

**Figure 4:**
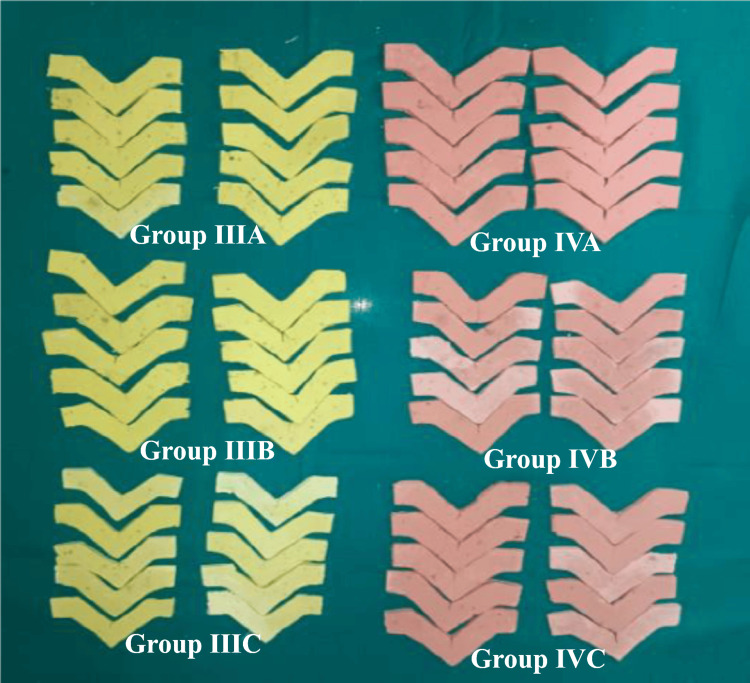
Samples (tear strength)

For the control group, 15 mL of water was used for mixing with 7 gm of Tropicalgin powder (Group IIIA), and 15 mL of water was used for mixing with 7 gm of Algitex powder (Group IVA) and placed into a sheet metal mold (Figure 4) with a V-shaped notch in the center and placed on a glass slab coated with Vaseline; pressure was applied to ensure an even thickness of alginate during gelation. After two minutes, the alginate specimens were retrieved from the mold and immediately subjected to the tear strength test using a universal testing machine (Figure 3) at a crosshead speed of 500 mm/min. The specimen's tear strength was calculated using the following equation: $\text{Tear strength} = \frac{\text{Load required to tear the sample}}{\text{Sample thickness}}$ [12].

For the experimental group, 2.2 mL of lignocaine (2%) and 12.8 mL of water were used for mixing with 7 gm of Tropicalgin powder (Group IIIB), and 2.2 mL of lignocaine (2%) and 12.8 mL of water were used for mixing with 7 gm of Algitex (Group IVB) and were placed into a sheet metal mold (Figure 4) with a V-shaped notch in the center and placed on a glass slab coated with Vaseline; pressure was applied to ensure an even thickness of alginate during gelation. For another experimental group, instead of lignocaine (2%), bupivacaine (0.5%) was used (Group IIIC, Group IVC), and the same procedures were followed. After two minutes, the alginate specimens were retrieved from the mold and immediately subjected to the tear strength test using a universal testing machine (Figure 3) at a crosshead speed of 500 mm/min. The calculation of the specimen's tear strength was performed using the following equation: $\text{tear strength} = \frac{\text{load required to tear the sample}}{\text{thickness of the sample}}$ [12].

Statistical analysis

Data analysis was performed using SPSS Statistics version 20.0 (IBM Corp. Released 2011. IBM SPSS Statistics for Windows, Version 20.0. Armonk, NY). The Shapiro-Wilk test was used to assess the normality of the data distribution. Because the data were normally distributed, parametric tests were utilized. A One-way ANOVA, followed by Tukey's post hoc test, was conducted to compare mean compressive and tear strengths among the groups. A p-value of < 0.05 was considered statistically significant.

## Results

Compressive strength

A one-way ANOVA revealed a highly significant difference in compressive strength between the control and experimental groups (p = 0.0001). For Tropicalgin, the control group (IA) exhibited a mean compressive strength of 0.405 MPa, which significantly decreased upon the addition of 2% lignocaine (IB: 0.356 MPa) and 0.5% bupivacaine (IC: 0.366 MPa). A similar significant reduction was observed in Algitex from the control (IIA: 0.385 MPa) to the experimental groups (IIB: 0.346 MPa; IIC: 0.355 MPa). Notably, the mean compressive strength for Algitex combined with 2% lignocaine (Group IIB) fell slightly below the 0.35 MPa minimum threshold stipulated by ADA Specification No. 18 (Table 1).

**Table 2:** Intergroup comparison of compressive strength * statistically significant (p < 0.05). Data analyzed using one-way ANOVA. Sample size n = 10 (100%) for all groups. SD: standard deviation, CI: confidence interval, ANOVA: analysis of variance

Group	n (n%)	Mean	SD	95% CI		p-value
				Lower bound	Upper bound	
IA – control (n = 10)	10 (100%)	0.4050	0.0299	0.3836	0.4264	0.0001*
IB – experimental (n = 10)	10 (100%)	0.3560	0.0150	0.3452	0.3668	
IC – experimental (n = 10)	10 (100%)	0.3660	0.0177	0.3533	0.3787	
IIA – control (n = 10)	10 (100%)	0.3850	0.0250	0.3671	0.4029	
IIB – experimental (n = 10)	10 (100%)	0.3460	0.0135	0.3363	0.3557	
IIC – experimental (n = 10)	10 (100%)	0.3550	0.0143	0.3447	0.3653	

Tear strength

Similarly, a highly significant difference (p = 0.0001) was observed in tear strength. Tropicalgin controls (IIIA) recorded 1.15 N/mm, which dropped significantly in the lignocaine (IIIB: 0.92 N/mm) and bupivacaine (IIIC: 0.94 N/mm) groups. Algitex controls (IVA) showed 0.83 N/mm, decreasing significantly in the experimental groups (IVB: 0.62 N/mm; IVC: 0.63 N/mm) (Table 2).

**Table 3:** Intergroup comparison of tear strength * statistically significant (p < 0.05). Data analyzed using one-way ANOVA. Sample size n = 10 (100%) for all groups. SD: standard deviation, CI: confidence interval, ANOVA: analysis of variance

Group	n (n%)	Mean	SD	95% CI		p-value
				Lower bound	Upper bound	
IIIA – control (n = 10)	10 (100%)	1.1500	0.2273	0.9874	1.3126	0.0001*
IIIB – experimental (n = 10)	10 (100%)	0.9200	0.1549	0.8092	1.0308	
IIIC – experimental (n = 10)	10 (100%)	0.9400	0.1577	0.8271	1.0529	
IVA – control (n = 10)	10 (100%)	0.8300	0.1059	0.7542	0.9058	
IVB – experimental (n = 10)	10 (100%)	0.6200	0.1549	0.5092	0.7308	
IVC – experimental (n = 10)	10 (100%)	0.6300	0.1159	0.5471	0.7129	

## Discussion

Impressions in dentistry play a very important role in the fabrication of indirect restorations, a fact that is often overlooked [13]. Alginate is the most commonly used impression material in dentistry. It is particularly well-suited for use as an elastic impression material, with many advantages, including ease of manipulation, lower cost, hydrophilicity, and patient comfort [2].

Pharmacological and non-pharmacological therapies are used to prevent the gag reflex. Gagging can be treated pharmacologically with either peripheral- or central-acting medications [14,15]. The use of local and topical anesthetics is believed to be an effective means of reducing excessive gagging [16].

Administration of local anesthetic agents around the posterior palatine foramen and the glossopharyngeal nerve block technique was also effective in patients with exaggerated gag reflexes. The deposition of local anesthetic agents may be impossible because they can provoke gagging and result in inaccurate impressions due to tissue distension [9]. To address these limitations, alginate impressions were attempted with the inclusion of local anesthetic agents, and the study conducted by Kamran and Qamar reported that the addition of local anesthetic agents (1.8 mL) to alginate could effectively reduce the excessive gag reflex in patients while making impressions. They also reported that the mean gag reflex score decreased significantly to 1.20 (according to the gag severity index) from 3.33 (before impression) after mixing the local anesthetic agents with alginate [17].

Similarly, Hussain et al. reported that mixing a single cartridge (2.2 mL) of lignocaine with irreversible hydrocolloid significantly controlled the gag reflex in 47.5% of patients with severe gag reflex and 52.5% with moderate gag reflex. In contrast, adding two cartridges of lignocaine to the hydrocolloid reduced the number of patients with severe gag reflex by 60% and with moderate gag reflex by 40% [18].

Compressive strength

A minimum compressive strength of 0.35 MPa for alginate impression material has been recommended in ADA Specification No. 18 [1]. In contrast to the findings of Qaisar et al., who reported an insignificant reduction in strength [10], our data showed a highly significant decrease in compressive strength (p = 0.0001) with the addition of 2% lignocaine and 0.5% bupivacaine.

However, from a clinical standpoint, the experimental groups mostly maintained compressive strengths near or slightly above the 0.35 MPa ADA threshold (e.g., Tropicalgin experimental groups measured 0.356 MPa and 0.366 MPa). The significant reduction in strength is likely due to the formation of an amide bond between the amino group of the local anesthetic agents (2% lignocaine) and the irreversible hydrocolloid's carboxyl group, which might have altered the crosslinking of the irreversible hydrocolloid's polysaccharide chain. This could also apply to 0.5% bupivacaine, used in this study, as both are amide local anesthetic agents [7].

Tear strength

The chemical composition, consistency, and manner of removal of a material influence its tear strength. The ability of a material to withstand tearing in thin interproximal areas and the depth of the gingival sulcus explains its tear strength. The tear strength is an important factor when an impression involves a mechanical undercut, and it needs sufficient bulk to resist tearing during removal [12].

Similar to compressive strength, our study observed a highly significant decrease in tear strength (p = 0.0001) following the addition of local anesthetic agents. The decrease in tear strength may indicate that covalent bonding between amide nitrogen and silicon dioxide (via O-Si-O) compromises the material's ability to withstand tearing [10].

Fourier transform infrared spectroscopy analysis

The functional groups in materials (gas, liquid, and solid) can be identified using Fourier transform infrared spectroscopy (FTIR), which employs a beam of infrared radiation. The absorption of infrared radiation was determined using infrared spectroscopy. Each molecular bond emits radiation, represented in the spectrum as % transmittance vs. wavenumber (cm⁻¹) [19].

Chemical bonds in a molecule can be identified by FTIR (Figure 4), which produces an infrared absorption spectrum. It can also identify unknown materials and determine the amount of components in a mixture. In the present study, FTIR analysis was done to check the presence of functional groups in irreversible hydrocolloid materials before and after the addition of local anesthetic agents [20].

The results for all samples (before and after setting) showed a peak for the OH group in the range of 3200 to 3550 cm⁻¹. In addition to this, the presence of the carbonyl (C = O) group is confirmed in the range between 1630 and 1700 cm⁻¹. This carbonyl (C = O) group suggests the presence of amide linkages, which show a peak in the same range as that of the OH groups. All the samples (before and after setting) showed a peak in the range of 2100-2250 cm⁻¹. This may correspond to carbon-carbon or carbon-nitrogen triple bonds (alkynes).

Additionally, the samples showed a peak in the fingerprint region, which ranges from 1400 cm⁻¹. The peaks in the fingerprint region were similar across all samples. In all samples (before and after setting), the C-F peak (aryl halide) was observed in the range of 1000-1400 cm⁻¹.

**Figure 5:**
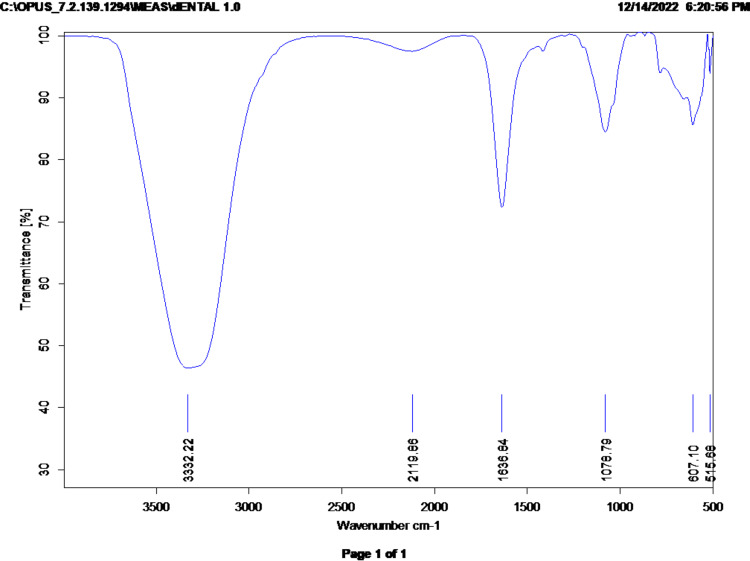
FTIR analysis - Group IA (before setting) FTIR: Fourier transform infrared spectroscopy

**Figure 6:**
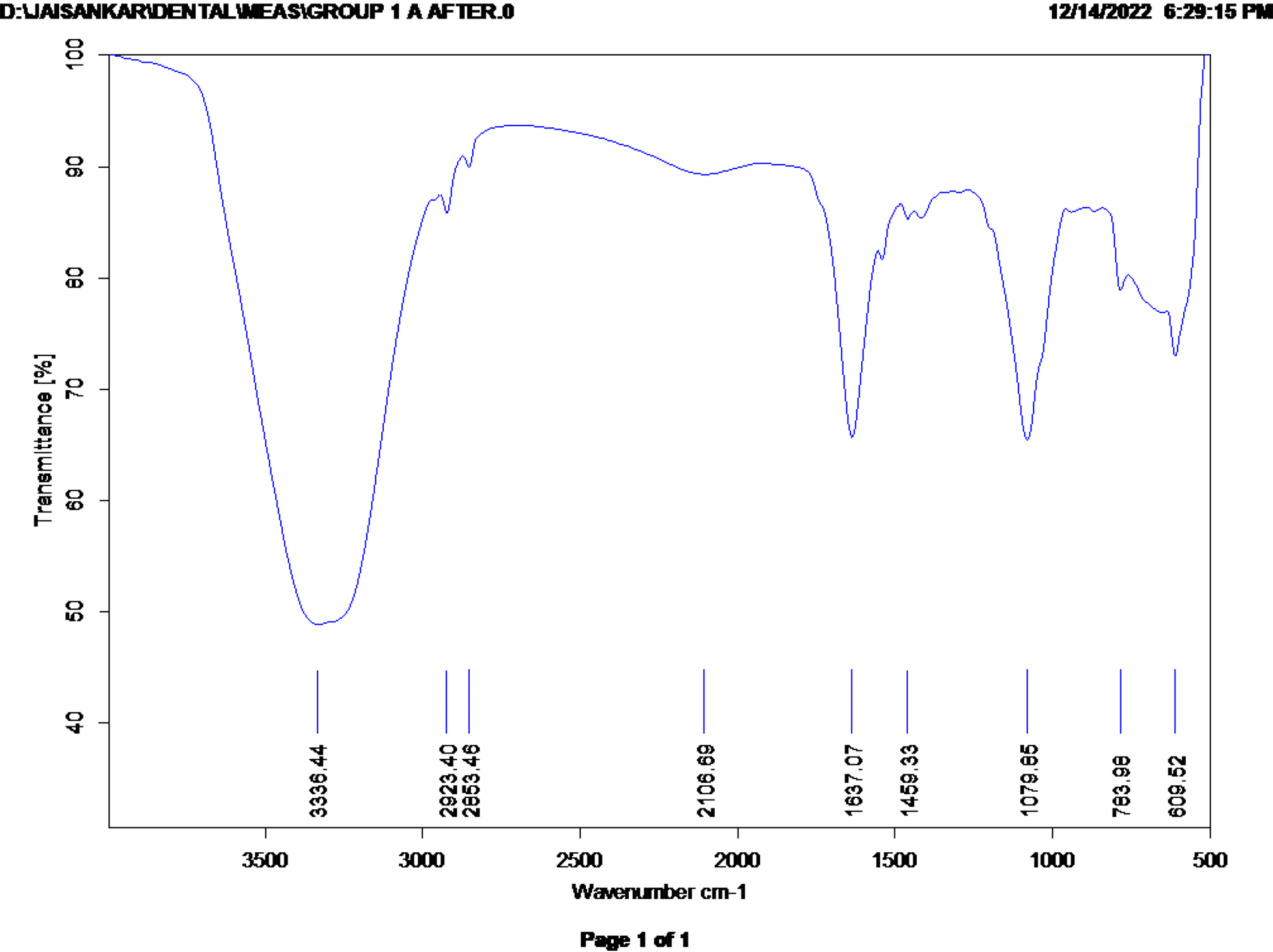
FTIR analysis - Group IA (after setting) FTIR: Fourier transform infrared spectroscopy

**Figure 7:**
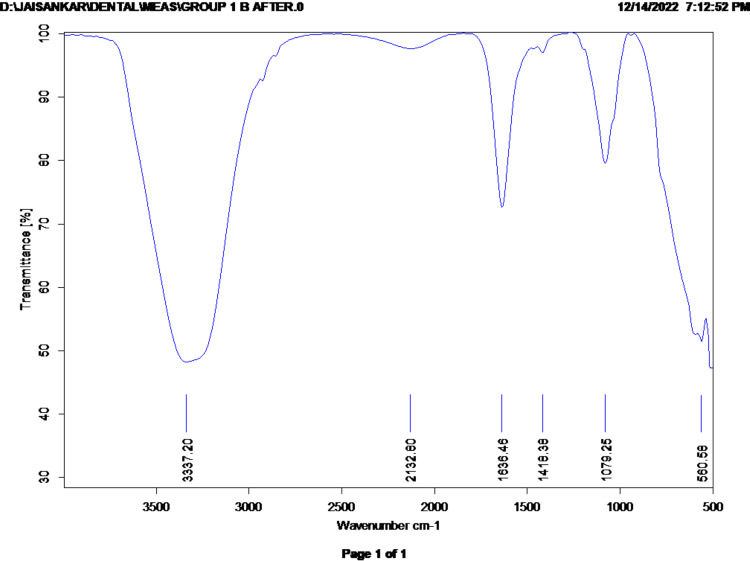
FTIR analysis - Group IB (after setting) FTIR: Fourier transform infrared spectroscopy

**Figure 8:**
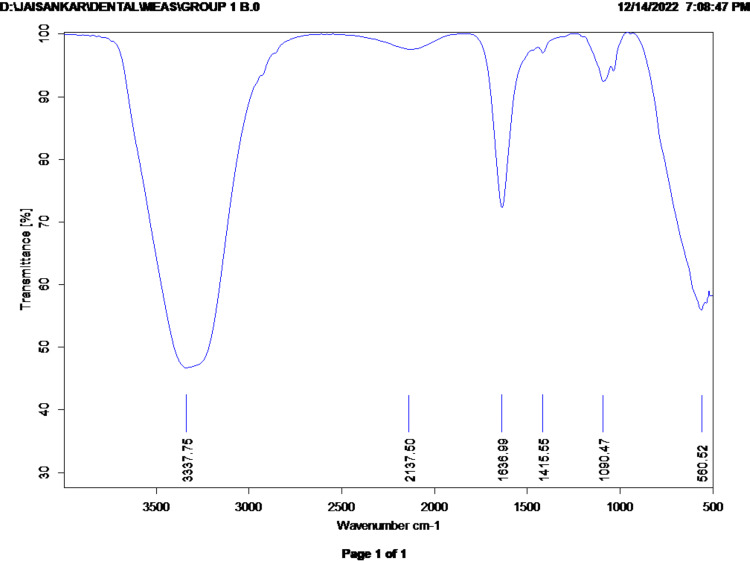
FTIR analysis - Group IB (before setting) FTIR: Fourier transform infrared spectroscopy

**Figure 9:**
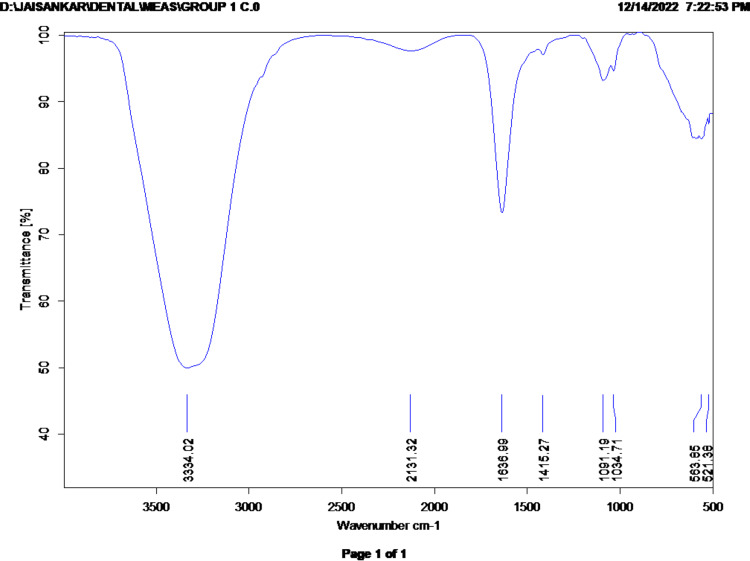
FTIR analysis - Group IC (before setting) FTIR: Fourier transform infrared spectroscopy

**Figure 10:**
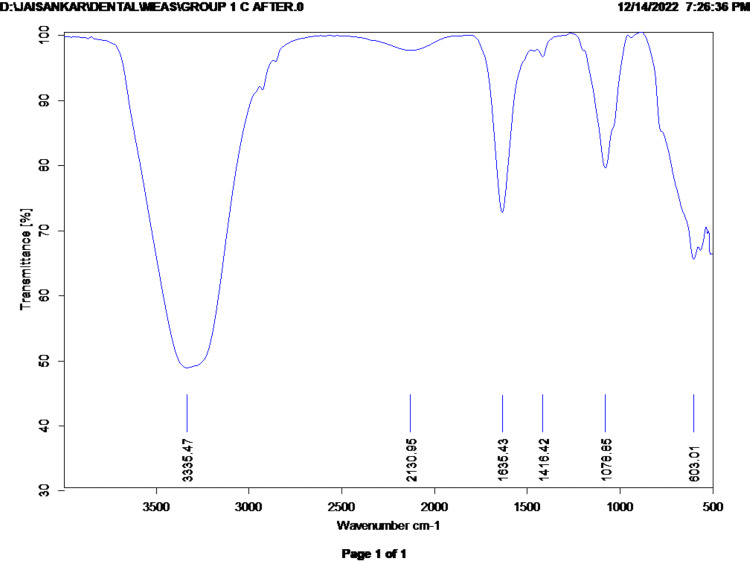
FTIR analysis - Group IC (after setting) FTIR: Fourier transform infrared spectroscopy

**Figure 11:**
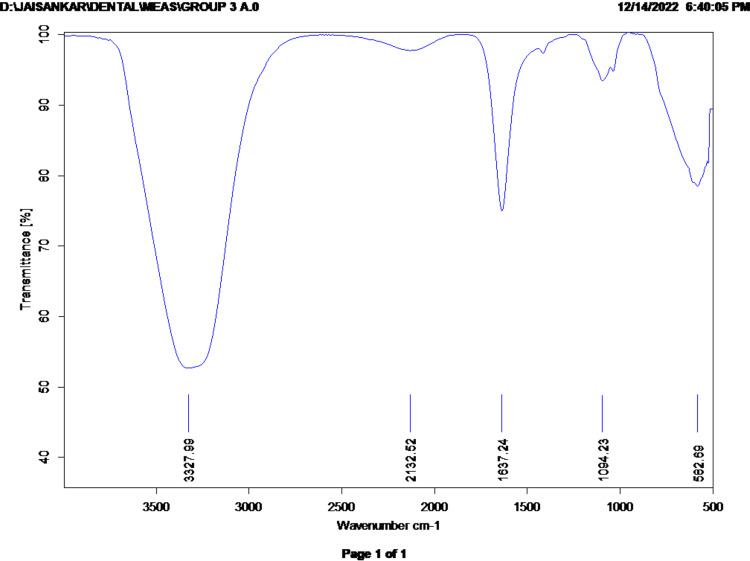
FTIR analysis - Group IIA (before setting) FTIR: Fourier transform infrared spectroscopy

**Figure 12:**
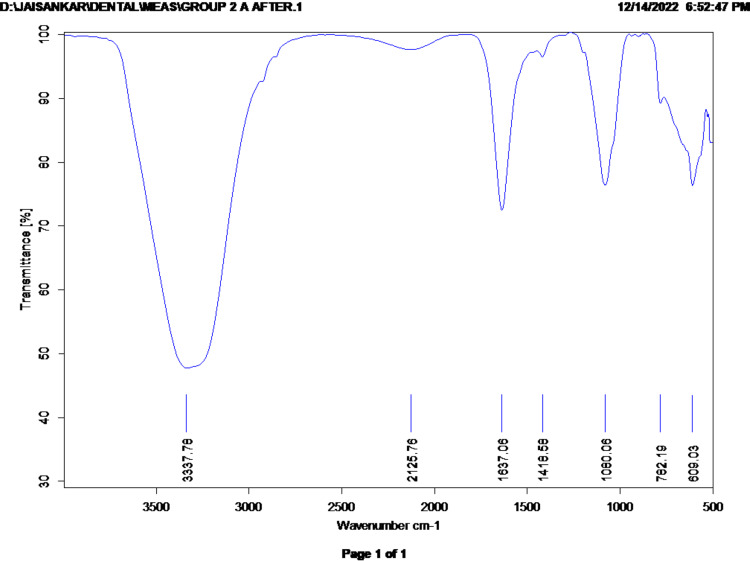
FTIR analysis - Group IIA (after setting) FTIR: Fourier transform infrared spectroscopy

**Figure 13:**
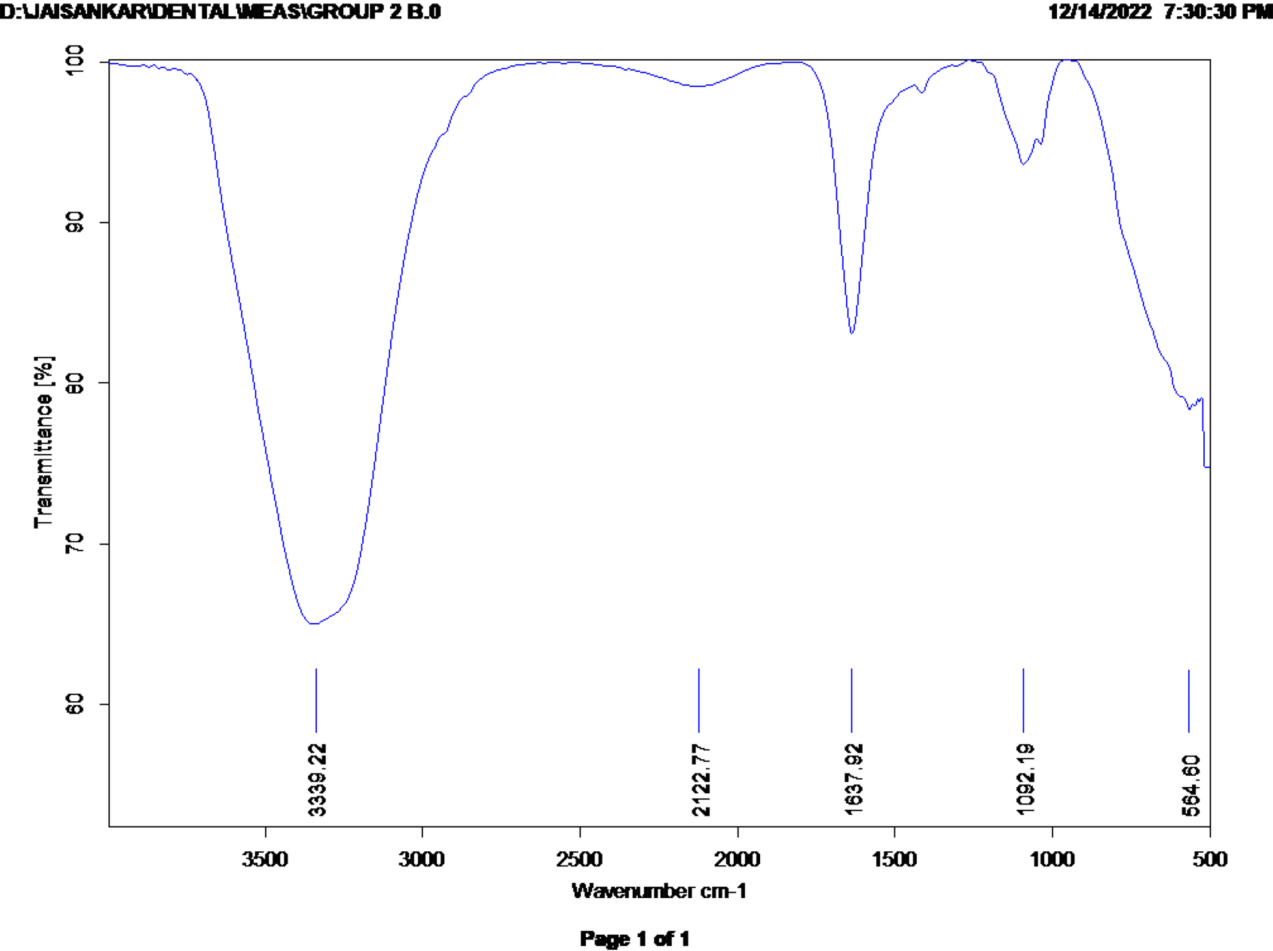
FTIR analysis - Group IIB (before setting) FTIR: Fourier transform infrared spectroscopy

**Figure 14:**
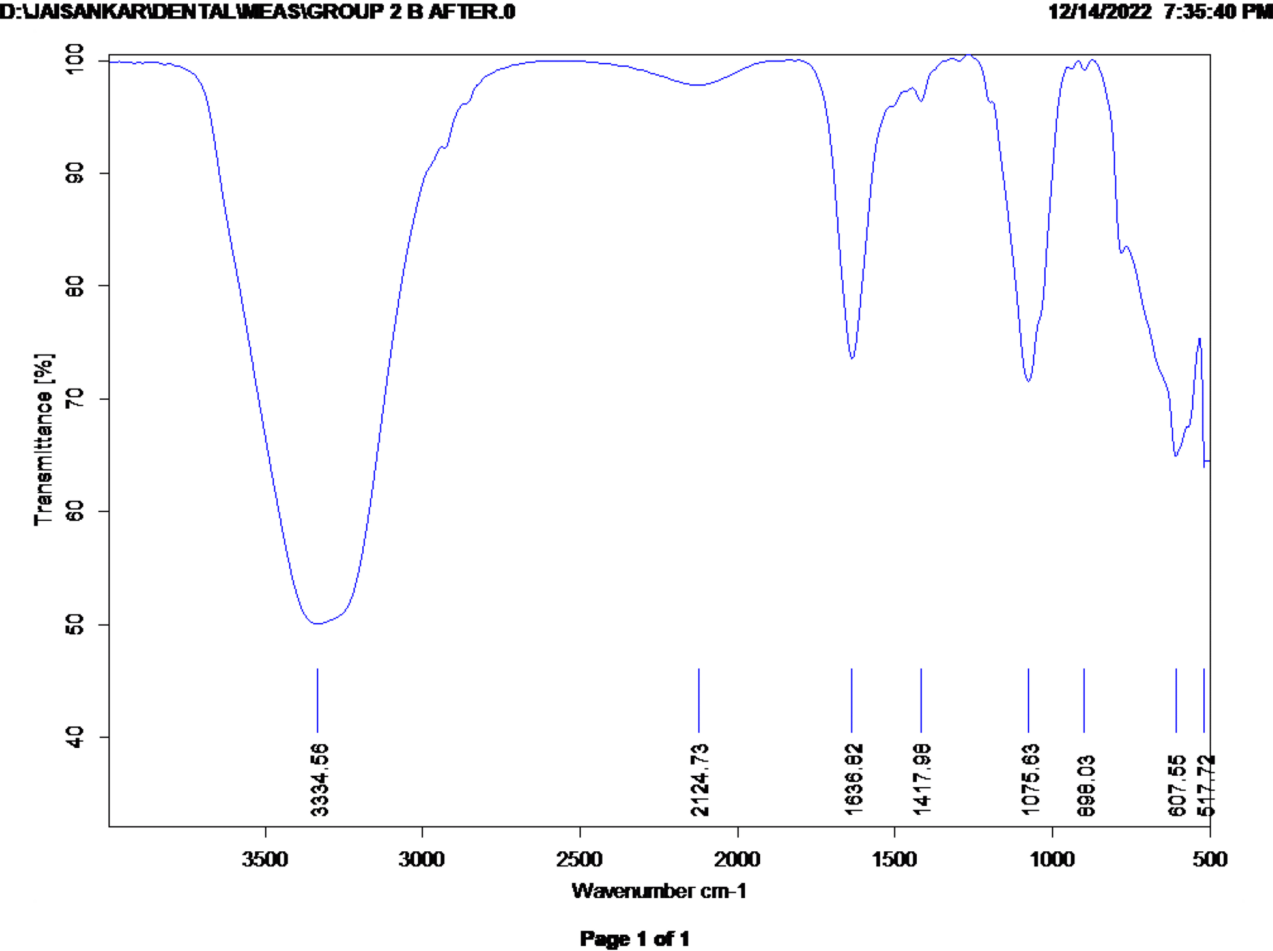
FTIR analysis - Group IIB (after setting) FTIR: Fourier transform infrared spectroscopy

**Figure 15:**
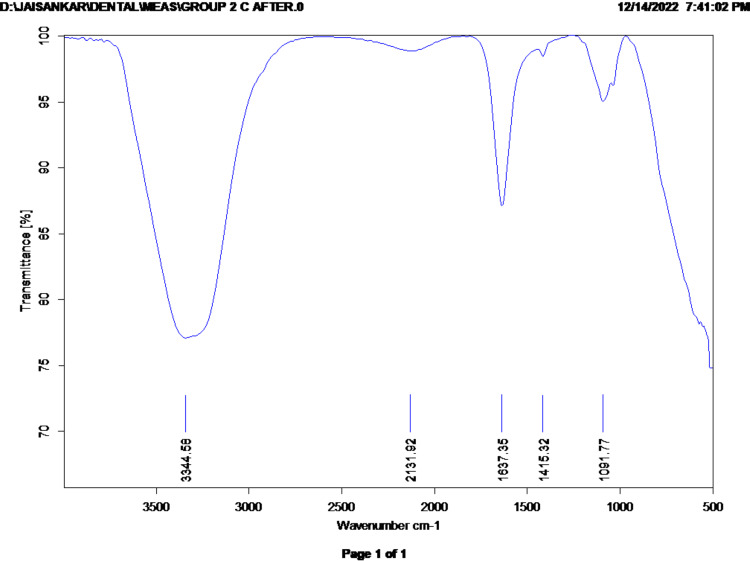
FTIR analysis - Group IIC (before setting) FTIR: Fourier transform infrared spectroscopy

**Figure 16:**
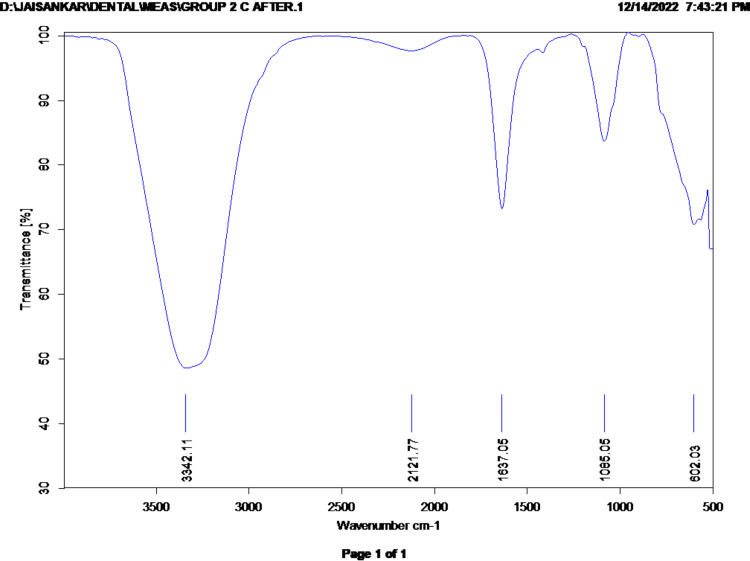
FTIR analysis - Group IIC (after setting) FTIR: Fourier transform infrared spectroscopy

The spectral patterns were similar, and there were no changes in peak positions or intensities upon incorporation of local anesthetic agents (2% lignocaine and 0.5% bupivacaine) into the irreversible hydrocolloid impression materials. Based on the FTIR results (Figures 5-16) and in line with findings from other studies, there is strong molecular-level affinity between the local anesthetic agents (2% lignocaine and 0.5% bupivacaine) and the irreversible hydrocolloid in all samples, both before and after setting.

Limitations of the study

As an in vitro laboratory study, the results cannot fully replicate the dynamic intraoral environment. Factors such as salivary flow, patient body temperature, and intraoral moisture are completely unaccounted for. Furthermore, using unstandardized manual pressure to compress the glass slabs against the molds introduces an operator variable that could inadvertently affect density and porosity across samples. While this study thoroughly tests compressive and tear strengths, it entirely omits other crucial properties governed by ADA Specification No. 18, such as setting time, working time, and elastic recovery, all of which are highly likely to change when structural water is substituted with an acidic anesthetic solution. Finally, it must be emphasized that retaining the ADA threshold in vitro does not guarantee adequate clinical performance or material stability inside the mouth.

## Conclusions

Within the limitations of this in vitro study, it can be concluded that adding 2% lignocaine and 0.5% bupivacaine to irreversible hydrocolloids significantly reduces their compressive and tear strengths, possibly due to physical dilution. While most experimental groups maintained compressive values near the clinically acceptable threshold of 0.35 MPa, certain material combinations (such as Algitex mixed with 2% lignocaine) fell below this threshold. Therefore, while this modification alters mechanical properties, it may still meet the minimum compressive requirements in select, controlled combinations. However, further in vivo clinical studies are warranted to confirm both the material's intraoral efficacy and its clinical safety for managing patients with a severe gag reflex.

## References

[1] Manappallil JJ (2010). Basic Dental Materials. Jaypee Brother.

[2] Cervino G, Fiorillo L, Herford AS (2018). Alginate materials and dental impression technique: a current state of the art and application to dental practice. Mar Drugs.

[3] Jain AR (2018). Gagging: a problem to prosthetic dentistry - review. Drug Invent Today.

[4] Prashanti E, Sumanth KN, Renjith George P, Karanth L, Soe HH (2015). Management of gag reflex for patients undergoing dental treatment. Cochrane Database Syst Rev.

[5] Hattab FN, Al-Omari MA, Al-Duwayri ZN (1999). Management of a patient’s gag reflex in making an irreversible hydrocolloid impression. J Prosthet Dent.

[6] Conny DJ, Tedesco LA (1983). The gagging problem in prosthodontic treatment. Part II: patient management. J Prosthet Dent.

[7] Malamed SF (2013). Handbook of Local Anesthesia. https://www.elsevier.com/books/handbook-of-local-anesthesia/malamed/978-0-323-07413-1.

[8] Moore PA, Hersh EV (2010). Local anesthetics: pharmacology and toxicity. Dent Clin North Am.

[9] Garg R, Singhal A, Agrawal K, Agrawal N (2014). Managing endodontic patients with severe gag reflex by glossopharyngeal nerve block technique. J Endod.

[10] Qaisar A, Hussain S, Yazdanie N, Khalid H, Khan AS (2019). Effect of lignocaine addition on the properties of irreversible hydrocolloid impression material. J Ayub Med Coll Abbottabad.

[11] Abdelraouf RM, Bayoumi RE, Hamdy TM (2021). Effect of powder/water ratio variation on viscosity, tear strength and detail reproduction of dental alginate impression material (in vitro and clinical study). Polymers (Basel).

[12] Fayaz A, Noori A (2016). Evaluation of tear strength of two types of Iralgin and its comparison with similar alginate impression material. J Dent Sch.

[13] Manar J (2018). Alginate as impression material. Int J Appl Dent Sci.

[14] Dickinson CM, Fiske J (2006). A review of gagging problems in dentistry: 2. Clinical assessment and management. S Afr Dent J.

[15] Ramsay DS, Weinstein P, Milgrom P, Getz T (1987). Problematic gagging: principles of treatment. J Am Dent Assoc.

[16] Forbes-Haley C, Blewitt I, Puryer J (2016). Dental management of the ‘gagging patient-an update. Int J Dent Health Sci.

[17] Kamran M, Qamar R (2016). An easy and effective way to reduce gag during orthodontic impression recording. Pak Orthod J.

[18] Hussain S, Batool S, Yazdanie N (2016). Gag reflex: not a dilemma anymore. Biomedica.

[19] Khan SA, Khan SB, Khan LU, Farooq A, Akhtar K, Asiri AM (2018). Fourier transform infrared spectroscopy: fundamentals and application in functional groups and nanomaterials characterization. Handbook of Materials Characterization.

[20] Thermo Nicolet Corporation (2001). Thermonicolet. Introduction to Fourier transform infrared spectroscopy. Introduction to Fourier Transform Infrared Spectrometry.

